# Opportunities and challenges for paediatricians requesting funded genomic tests for children

**DOI:** 10.1038/s41431-025-01864-3

**Published:** 2025-05-07

**Authors:** Belinda Dawson-McClaren, Melissa Martyn, Jessica Ince, Alli Jan, Natasha J. Brown, Michael C. Fahey, Erin Crellin, Clara Gaff

**Affiliations:** 1 Melbourne Genomics Health Alliance, Melbourne, VIC Australia; 2 Murdoch Children’s Research Institute, Melbourne, VIC Australia; 3 Department of Paediatrics, University of Melbourne, Melbourne, VIC Australia; 4 Victorian Clinical Genetics Service, Melbourne, VIC Australia; 5 Department of Paediatrics Monash University, Melbourne, VIC Australia

**Keywords:** Social sciences, Genetic testing, Health services, Medical genomics

## Abstract

Genomic diagnosis for children with childhood syndromes can inform treatment, management and reproductive planning. Shortages in the clinical genetics workforce mean that practices are changing, with paediatricians likely requesting initial genomic investigations and clinical genetics services reserved for particularly complex cases or post-test genetic counselling. In Australia, paediatricians can request funded genomic testing for patients, yet ordering rates are less than a quarter predicted. Using a theory-informed approach, we aimed to understand barriers and enablers to general paediatricians’ practice as the essential first step in developing complex interventions to support clinical practice. Maximum variation sampling was used to invite general paediatricians to complete a semi-structured interview. The interview guide used a process map of the steps involved in ordering funded genomic tests, with questions addressing the components of the Capability, Opportunity, Motivation – Behaviour (COM-B) model and the Theoretical Domains Framework. Twenty-six general paediatricians with diverse practice experience participated. Paediatricians described barriers related to capability, opportunity and motivation. Intuitive strategies general paediatricians suggested to overcome barriers aligned with theoretical strategies to implement practice change. These included: raising awareness using avenues paediatricians already access for clinical information; providing opportunities for experiential learning to build on foundational knowledge; having practical resources in one easily accessible location; family-friendly information materials to share with patients. This study provides evidence to inform a roadmap of strategies to effectively support general paediatricians in incorporating genomics into their practice and ultimately delivering faster, more equitable genomic medicine.

## Introduction

Adopting emerging technologies into healthcare only happens with significant time and effort. It takes on average 17 years for this to occur, with many innovations failing to even bridge the gap [1]. Genomic medicine was defined in 2013 by Manolio and colleagues as “using an individual patient’s genotypic information in his or her clinical care” [2]. In their definition, they anticipate, and it has eventuated, that genomic medicine is an area of practice in which emerging technologies are drivers of clinical change. The term genomic medicine can be used to describe the processes and care that surround, and include, when a test is requested such as consent and information discussions, and result return. The diagnostic yield of genomic testing (defined herein as genome-wide sequencing tests such as exome sequencing) can be 10–60%, depending on the patient group [3–5]. Where clinical utility is demonstrated, there are mechanisms by which genomic testing can be funded to improve patient access [6]. This has been the case in Australia: from May 2020, a rebate became available through the national Medicare^1^ benefits scheme for genomic tests requested directly by paediatricians or clinical geneticists for childhood syndromes with specific neurodevelopmental and syndromic presentations [7, 8]. Enabling general paediatricians to request funded genomic tests via whole exome or genome sequencing and analysis was anticipated to enhance patient access to timely, accurate diagnoses.

Since commencement, however, use of the Medicare rebates has been only a quarter of the predicted use [9]. It is complex to introduce genomic medicine into health systems and the absence of a targeted, conscious action plan to support general paediatricians to adopt requesting funded genomic tests as part of their practice has likely led to this low activity [10]. Similarly, despite guidelines in the US and Canada recommending genomic testing for various paediatric presentations [11], clinicians report low levels of understanding, confidence, and utilisation of available services [12–14].

A sophisticated approach to understanding barriers and enablers to the behaviour is required to develop and evaluate interventions to address complex problems [15]. Theory-informed approaches enable a more complete understanding of barriers and enablers, and help identify targeted implementation strategies. However, such approaches remain relatively rare in the context of implementing genomic medicine. To the best of our knowledge, there have been no comprehensive, theory-informed investigations of factors affecting the adoption and use of funded genomic testing in outpatient paediatric settings. This study used the Capability, Opportunity, Motivation – Behaviour (COM-B) model and Theoretical Domains Framework (TDF) as guiding approaches to explore general paediatricians’ behaviour and influences on behaviour related to genomic testing [16, 17]. Our findings lay the groundwork for developing effective strategies to support paediatricians to incorporate genomics into their practice and ultimately, deliver faster, more equitable genomic medicine.

## Subjects and methods

### Participants

Semi-structured telephone interviews were conducted with general paediatricians in Victoria, Australia and included participants practising in metropolitan Melbourne and regional Victoria. Paediatricians were in public and/or private practice and were not paediatric sub-specialists such as paediatric neurologists or nephrologists. The term paediatrician used in this study describes general paediatricians.

A purposive sampling strategy, for maximum variation, was used; particular attention was paid to sampling from regional and private practice settings [18]. Paediatricians were contacted by direct email to [1] inform them about the study and [2] invite them to participate in an interview via these avenues:

- contacts and networks of the authors
- publicly available email addresses of paediatricians
- attendees of an online webinar focussed on exome testing delivered by the Melbourne Genomics Health Alliance [19]

Verbal consent was obtained at the start of the interview. A digital recorder was used to capture audio data, the digital audio file was transcribed verbatim, the final transcript was checked against the audio for accuracy prior to analysis. Interview participants were asked to pass on the study information to colleagues in a snowball sampling approach.

### Interview structure and design

An interview guide was developed by the project team using components of the COM-B and TDF to provide a structured approach to understanding influences of behaviour. Key, interrelated components of these frameworks include the motivations, psychological and physical capacity, opportunity including financial, time, and resource constraints. A process map approach was included in the interview guide to seek responses from paediatricians about barriers experienced or anticipated when identifying suitable patients, completing test request forms, consulting with a clinical geneticist, explaining testing & obtaining consent, receiving result reports, and returning results to patients [20]. All interviews were conducted by BDM, an experienced qualitative researcher who is not a genetic health professional or a general paediatrician. The interviewer was unknown previously to the interviewees.

To contextualise responses about barriers, paediatricians were also asked general questions about their area of practice and their experience of requesting or referring patients for genomic testing. Participants were asked to suggest strategies and solutions around reducing and overcoming barriers to ordering.

Table 1 describes, in context, the COM-B components and TDF domains components.

**Table 1:** COM-B components defined in the context of funded genomic tests, with mapped domains from the Theoretical Domains Framework.

COM-B component	Definition in context	TDF domains mapped
Capability	Assesses psychological and physical capacity of paediatricians to order funded genomic testing and its associated tasks. Factors such as knowledge gaps, the complexity of interpreting genomic data, and limited access to genetic specialists may hinder the effective utilisation of funded genomic testing	Knowledge; Skills (psychological and practical); Memory, Attention and Decision Processes; Behavioural Regulation
Opportunity	Assesses environmental and social factors that influence behaviour. Challenges such as financial constraints, time limitations, and the availability of resources may impact paediatricians’ ability to offer this diagnostic option to their patients	Social Influences; Environmental Context and Resources
Motivation	Assesses attitudes towards funded genomic testing, perceived value for patients and families, and the level of support and encouragement from peers and healthcare institutions	Social/Professional Role and Identity; Beliefs about Capability; Optimism; Beliefs about Consequences; Intentions; Goals; Reinforcement; Emotion

### Data analysis

Three researchers coded interview transcripts to the domains of the TDF: BDM coded all transcripts and supervised co-coding by two research assistants using a codebook. Two researchers coded each transcript independently in NVivo 14 (Lumivero, 2020). Barriers and exemplar quotes were discussed in regular research meetings involving BDM, MM, EC, and CG.

## Results

Eighty-five paediatricians were contacted regarding participation in the study; 54 of these were emailed directly by a study author or a contact of a study author; 19 were ‘cold-call’ emailed using publicly available information; 12 were participants of a webinar who expressed an interest in the study. Interviews were completed with 26 paediatricians, with interview duration ranged from 33–46 min. Sixteen practiced in metropolitan Melbourne and ten in regional sites across the state. Most participants (85%, *n* = 22/26) had not previously ordered a funded genomic test. Nine paediatricians were in private practice only (six metropolitan, three regional), eight practiced in a public hospital with an in-house clinical genetics service (all metro), and nine worked in a public hospital with a visiting clinical genetics service (two metropolitan and seven regional).

### The practice of genomic medicine by paediatricians in Victoria

In this study, paediatricians described themselves as generalists. Their patients experienced myriad and often complex medical care needs, so requesting funded genomic testing requires clinicians to learn and undertake new tasks in their practice. They referenced similar experiences with the introduction of chromosomal microarrays into their regular practice, which despite the steep learning curve is now a familiar and common part of practice for paediatricians in Australia.

Participants were asked about the advantages of a paediatrician ordering the test; most initially responded in general terms about the overall benefits of genomic tests and had little to say specifically about the advantages of testing being requested by them rather than a clinical geneticist.

When probed further, paediatricians identified benefits for their patients if they ordered the funded genomic tests to be: eliminating long waiting times at genetic services for referral appointments; and, their patients being able to attend a genetics appointment with test results to then have the opportunity to discuss in more detail with a clinical geneticist.

The potential of funded genomic testing to provide answers for families, reduce other diagnostic testing and make care management decisions was a driver of positive attitudes:

*I’ve just seen the benefit in terms of having a one-off genetic test for someone with a complex problem. If you can nail the diagnosis early in that journey of seeking a diagnosis you can cut out so many tests that would have been unnecessary and yielded nothing and then you can focus on targeted investigations and targeted management. If I think about the patients and who I would still like to do some of these tests, but they haven’t been eligible, it can be years of anxiety. (Paediatrician1)*

Paediatricians described having reservations about requesting funded genomic testing for their patients. They wanted to ensure that their patients received high-quality, equitable care when a genomic investigation was undertaken; they expected that a specialised genetic service and genetic health professionals had the knowledge and skills to do this better than they did. In their view, genomic tests were generally not urgent. Therefore families waiting for genetic service referral appointments was perceived as a reasonable trade-off given that they thought their patients would receive better, specialised care with a referral. Paediatricians described their low confidence, and skills, across all aspects of genomic medicine which many attributed to inexperience and anticipated that over time as they began to order genomic testing they would grow in confidence and competence.

Few paediatricians had ordered genomic testing for their patients at the time of interview, and those who have referred patients to a genetic service described desiring greater insight into what was discussed with patients at those appointments. Some gave examples in which a result had been given to their patient in a genetic service and in most cases the impact on treatment and management was low from the paediatrician’s perspective. When probed, paediatricians described how a genomic test result leading to a diagnosis was beneficial to families by: ‘providing certainty’, ‘parents just want to know’, ‘have a named diagnosis to access support services’, ‘connecting with other families’.

### Barriers identified by paediatricians

Paediatricians were asked about their experiences (or anticipated experiences) requesting funded genomic testing and patients who might be appropriate for a genomic test. This discussion identified barriers (actual or perceived) and factors that would influence their use of genomic testing. The results are organised using the components of the COM-B with domains of the TDF shown with underline formatting below.

#### Barriers related to capability

Given their limited direct experience ordering funded genomic testing at the time of the interview, it is unsurprising that participants described experiencing cognitive overload (memory, attention, and decision processes domain), which impaired their recall of information such as specific funded testing criteria for establishing patient eligibility. They also cited a need for more procedural knowledge (knowledge domain) related to accessing and completing test request forms.

*Knowing whom to test…if you look at what Medicare says are indications to do whole exome sequencing. I can’t remember, there was something that I thought, I don’t really understand what they’re trying to say there…It’s working out that grey area. (Paediatrician3)*

*I just don’t know enough about it to even consider doing it at the moment. I don’t know who’s eligible, how I do it, what’s involved, really, everything. (Paediatrician7)*

Paediatricians identified that they also lacked or had limited skills (skills domain) to talk to families about genomic tests and obtaining informed consent, to provide accurate and complete information on test requests, and to talk to families about results.

*I can’t at the moment see myself doing that, because the difficulties from my point of view are that all the consent bits and pieces that go with that are pretty involved, and I don’t know that I would … That’s one of the things that bothers me, all the consent things. (Paediatrician16)*

*Being able to competently talk about what implications this new diagnosis has for families, it’s a complex area, so that’s a major barrier. (Paediatrician8)*

#### Barriers related to opportunity

The paediatrician must consult with a clinical geneticist to request funded genomic testing under the Medicare rebate. For some interviewees, this was an immediate barrier as they did not know how they would make contact for such a consultation and lacked social support (social influences domain) due to the absence of these interpersonal professional connections.

*I wouldn’t actually know how I would go about, who I would speak to and how I would speak to them, which would be a barrier to me to do it. (Paediatrician7)*

Due to the perceived complexity of the discussions with families, paediatricians identified an environmental stressor (environmental context and resources) that impeded their opportunity, stating that their appointment times would be too short to cover everything adequately.

*If there were longer appointments, so if there were a separate rebatable item for genetic counselling that general paediatricians could [use] … I’m fighting inside against the kind of sinking feeling of ‘oh gosh, there’s another thing that we have to do now’. (Paediatrician17)*

#### Barriers related to motivation

The quote above also indicates an emotional response paediatricians experience that would negatively influence their motivation to order funded exome testing.

Commonly, participants spoke of having limited professional confidence (social/professional role and identity) in determining clinical eligibility such as being able to identify and describe dysmorphic features, to make a diagnosis.

*I’m not sure I have the expertise. I always think there might be something obvious that I’m missing, based on dysmorphology, and so I refer on [to a clinical genetics service] to see if, based on dysmorphology, there may be an obvious diagnosis (Paediatrician7)*

Having limited professional confidence highlighted a tension for participants, who reflected on their perception of their professional role (social/professional role and identity). They emphasised the generalist approach of paediatricians to managing children’s myriad health concerns, contrasting this with their perceived need for specialist skills to order and discuss genomic tests. For some, this was a sense of being out of their comfort zone; others held the view that genomic testing such as this should remain within the remit of a clinical genetics service.

*A lot of paediatricians have a natural caution about not stepping outside their skillset (Paediatrician2)*

*As general paediatricians, we see very complex patients who usually come for a review with a whole host of issues…the benefit of having a separate speciality is, they are just looking at the genetics. We rarely have the ability to dedicate an appointment to just the genetics because they’ll hijack it with their sleep or their continence or all the other things that come up. (Paediatrician7)*

Paediatricians expected difficulties in gathering and reporting all the clinical and familial information required to order testing (perceived behavioural control (beliefs about capability)).

*I’m worried the forms are really detailed in terms of the examination findings and all the parental information. I’m worried that it’s going to be extremely arduous to get all that information and to do it justice. (Paediatrician10)*

Of the few participants who had experience ordering funded tests, all were yet to directly receive results for a patient at the time of the interview. Nonetheless, participants drew on their prior experiences with microarray testing to describe having outcome expectancies (beliefs about consequences) that the test result will be uninformative. This contributed to feeling unconvinced of the value of the testing and influenced their lack of motivation to incorporate such testing into practice. There were two expected outcome scenarios described by participants: that no pathogenic variants will be reported (low yield); or a variant is reported yet the impact on treatment and management is minimal and therefore there is little value add to a clinical diagnosis.

*It’s a child with unexplained intellectual disability, and the [chromosomal] micro[array] is normal, I would have thought that the yield would be quite low if anything. (Paediatrician6)*

*A group like autism…well they’ve already got autism which gets them NDIS [access to the National Disability Insurance Scheme] and teacher’s aides and things, and therapy. So adding ‘you have a 6q19.3 deletion’, how does that help? (Paediatrician11)*

### Strategies to reduce or remove barriers

Paediatricians were asked to think of ways to overcome or reduce barriers to facilitate the behaviour of requesting funded genomic testing, when appropriate, for their patients.

#### Awareness raising

Paediatricians suggested raising awareness about the availability of funded genomic testing through newsletters and email lists they already subscribe to, presentations at health service department seminars and meetings, and websites of organisations such as laboratories performing the testing, medical colleges, and specialised associations, such as the Neurodevelopmental and Behavioural Paediatric Society of Australasia (NPBSA).

*I went to an afternoon workshop a couple of years ago… it was extremely good. I’ve attended grand rounds and paediatric updates and things like that whether it be face-to-face or webinar type things. (Paediatrician1)*

#### Education and knowledge building

Paediatricians had an appetite for educational activities such as webinars or education presentations at conferences. The opportunity to put learning into practice, experiential learning, was discussed to consolidate learning and further develop skills and confidence. Paediatricians thought it could be helpful to watch videos of genetic specialists talking about, for example, test consent; they also suggested co-consultation opportunities, which would provide them with experiential learning of how a genetic specialist would convey information to families or assess patient eligibility.

*The most useful thing is doing a role play, but all of us hate role plays, so the next thing is watching it and at least, you do absorb a lot with how people do it by watching… Having the ability to sit in with a geneticist or that joint consultation where a geneticist takes the lead, but you watch how they do it, for two or three, would be really helpful. (Paediatrician7)*

#### Practical supports

Paediatricians suggested that it would be helpful to have a checklist and further guidance on assessing their patients against eligibility criteria for funded testing.

*A checklist to check-in with the kid who presents with autism or the kid who presents with ADHD or the kids who presents with ID, to go through and say “Have they had their fragile X, have they had their microarray?” Because a lot of that happens intuitively in my head…then you think about changing that or adding in a new investigation, one of the ways to do that is checklist type medicine. (Paediatrican2)*

Some further ideas were for developing clinical practice guidelines and resources that paediatricians can access and refer to when encountering a patient who might benefit from a genomic test. Aligned with this desire for clear and relevant information was a requirement to have it available in one place.

*If there is like a written source then we can always go back and refer…Because we would, like because it’s all the information is in one place rather than like, you know, trying to search for it, on different sites. (Paediatrician15)*

To address the barriers related to strengthening professional networks and connections, for example with a clinical geneticist for consultation as required by the eligibility criteria, or for a broader range of support needs, paediatricians suggested an on-call geneticist accessible by a common phone number or a compilation of current contact details readily available.

*It only works if you actually can get onto a geneticist you know, it would be really helpful if we had a list, you know these are phone numbers, these are the people you can call, this is there’s a geneticist on call that you can get onto to ask a question. That would be really helpful because in private (practice), unless you’ve got someone on your list you don’t know where to start. (Paediatrician14)*

#### Resources suitable for sharing with families

Family-friendly resources were desired by paediatricians with the anticipation that these would assist with the communication of information and may contribute to more efficient conversations during the short appointments.

*I know that there are factsheets and things around about exomes…this can be a challenging area to explain to families, especially if you’re seeing a family who’s got English as a second language. A lot of my kids have a low IQ, the parents unfortunately have a low IQ as well and so actually trying to explain to them what the test involves…some sort of YouTube things that they could watch to explain what the test is. (Paediatrician2)*

In summary, the barriers discussed by participants could be described as being at the individual level such as beliefs and perceptions of knowledge, skills and capability; and at the service level such as resources, professional connections and time in appointments. Proposed strategies to overcome these barriers were not targeted at one barrier only, rather were interconnected with strategies impacting multiple barriers. The strategies included: instructive learning (making available clear, accurate information in an easy to find place), experiential learning (through observation and co-consultation opportunities), and provision of resources to support efficient practice such as family friendly materials and easy test request processes.

## Discussion

This study describes paediatricians’ experiences and views on requesting funded genomic testing in outpatient paediatric settings. Through engagement with paediatricians in metropolitan and rural settings, rich insights illustrate the underutilisation of these funded tests [9]. While it may have been expected that removing a significant barrier such as funding would increase utilisation, this study describes important barriers that persist and influence behaviour despite the availability of funded tests. Barriers occurred at the individual level of the clinician and at the service level, such as appointment length and access to a clinical geneticist for consultation. Specifically, the interviews revealed the competing priorities faced by paediatricians providing care for their patients in appointments that already feel too short to cover the myriad topics important to families. Funded genomic testing was perceived as likely beneficial to the families, and paediatricians anticipated genomic testing would increasingly become part of their practice. At present, where barriers exist or are anticipated, paediatricians described that genomic testing was a low priority in their practice and therefore they were likely to not discuss it or request it for their patients.

Shortages in the clinical genetics workforce [21, 22] mean that paediatricians are likely to be requesting initial genomic tests, with clinical genetics services reserved for particularly complex cases or post-test genetic counselling for recurrence risk and reproductive planning. Yet, reported practice for paediatricians at the time of this study was to primarily refer their patients to specialist genetic services when there was an indication for genomic test. There is a shift towards non-genetics professionals initiating such investigations, exemplified by the availability of funded tests through Medicare [23]. In the interviews, paediatricians described a rationale for resisting this shift as being the perception of the superiority of genetic services in delivering patient education and counselling for genomic tests.

Strategies that are effective in addressing barriers and supporting paediatricians to incorporate genomics in their practice are crucial. By using the COM-B and TDF to elicit barriers, we can consider appropriate implementation strategies that have evidence of effectiveness to support implementation new practices, as complied by expert consensus [24].

Paediatricians identified several individual-level factors affecting their ability to order funded genomic tests. Key among these were knowledge/skill limitations which are likely to be amenable to direct interventional improvement through education provision, a solution paediatricians specifically noted would involve resources being provided in one, easy to find place. Education as an implementation strategy is reported in the compilation list published by Powell et al. (2015) with a description of: “develop and format manuals, toolkits, and other supporting materials in ways that make it easier for stakeholders to learn about the innovation and for clinicians to learn how to deliver the clinical innovation”. Education is an often-proposed avenue to support behaviour change; whilst it makes some impact on filling a knowledge gap, it is insufficient alone [25, 26].

With mainstreaming of genomic medicine emerging in many medical settings, the barrier of clinicians being time poor is commonly experienced. While more time may not be possible to generate, supporting clinicians to be efficient, and preparing patients and families to discuss genomic tests may mean that the barrier of being time poor is reduced. Digital tools and resources for patients have facilitated efficiency in the context of genetic services; a recent review found that use of these were primarily for provision of education/information, and to support decision making [27]. Decision aids have been developed and tested as a helpful way for parents to take in educational material and apply it to making a decision about genomic testing for their child [28]. Another approach, to support decision and consent about incidental results, is the Genomic AdvISER, created by Bombard and colleagues [29] which has been shown in a randomised clinical trial to reduce counselling time in a cancer genetic counsellor clinic session [30]. Further research would be needed to determine if such approaches would be feasible and acceptable in paediatric outpatient settings.

Experiential, active learning is established as an approach to learning new skills and adopting practices into healthcare [31, 32]. In this study, paediatricians expressed a desire for experiential learning, by observing a genetic specialist through videos, or co-consultation. This intuitively suggested strategy appears in the Powell et al. compilation list as “Shadow other experts” with a description of “Provide ways for key individuals to directly observe experienced people engage with or use the targeted practice chance/innovation” [33]. Medical specialists offered the opportunity to ’immerse’ in genomics previously described the experience as positively impacting their capability by developing skills in discussing testing with patients, understanding processes and strengthening multidisciplinary relationships with clinical genetic services [33]. However, the extent of immersion or shadowing experience required for competency remains unknown and may well be variable.

This study sought a wide range of perspectives from paediatricians and through extensive efforts for recruitment, was successful in obtaining a sample of 26 paediatricians with diverse geographical representation, but few participants had direct experience ordering funded genomic tests. We did not observe striking differences in the perspectives provided by paediatricians in different practice settings, suggesting that these barriers are commonly experienced or anticipated to be experienced. This study has not sufficiently captured views on barriers and enablers from paediatricians who have ordered genomic tests. Over time there would likely be a larger sample of paediatricians who have ordered funded genomic tests available to conduct a follow up study examining their experiences and remaining barriers. That follow up study would provide more insight and comparisons with this present study to inform development of strategies to address barriers. Nonetheless, understanding behaviour at an early stage in adoption provides a foundation for designing interventions to support change in practice. As paediatricians gain experience, factors impacting uptake of investigations are likely to evolve.

Through qualitative, robust exploration, this study has identified implementation strategies to help overcome barriers that could support paediatricians in requesting funded genomic tests for their patients. Situating the strategies with theoretical frameworks suggests that, if trialled, they would likely impact behaviour change. There is a need to develop and implement interventions that use these strategies to support paediatricians in requesting timely genomic tests for their patients.

## Data Availability

The datasets generated during and/or analysed during the current study are available from the corresponding author on reasonable request.

## Footnotes

1 Australian Government, Department of Health and Aged Care. Medicare Benefits Schedule - Item 73358.
